# Simple parametric survival analysis with anonymized register data: A cohort study with truncated and interval censored event and censoring times

**DOI:** 10.1186/1756-0500-4-308

**Published:** 2011-08-25

**Authors:** Henrik Støvring, Ivar S Kristiansen

**Affiliations:** 1 School of Public Health, Biostatistics, Aarhus University, Bartholins Allé 2, DK-8000 Aarhus, Denmark; 2 Institute of Health Management and Health Economics, University of Oslo, P.O. Box 1089, Blindern NO-0317, Oslo, Norway

## Abstract

**Background:**

To preserve patient anonymity, health register data may be provided as binned data only. Here we consider as example, how to estimate mean survival time after a diagnosis of metastatic colorectal cancer from Norwegian register data on time to death or censoring binned into 30 day intervals. All events occurring in the first three months (90 days) after diagnosis were removed to achieve comparability with a clinical trial. The aim of the paper is to develop and implement a simple, and yet flexible method for analyzing such interval censored and truncated data.

**Methods:**

Considering interval censoring a missing data problem, we implement a simple multiple imputation strategy that allows flexible sensitivity analyses with respect to the shape of the censoring distribution. To allow identification of appropriate parametric models, a *χ*^2^-goodness-of-fit test--also imputation based--is derived and supplemented with diagnostic plots. Uncertainty estimates for mean survival times are obtained via a simulation strategy. The validity and statistical efficiency of the proposed method for varying interval lengths is investigated in a simulation study and compared with simpler alternatives.

**Results:**

Mean survival times estimated from the register data ranged from 1.2 (SE = 0.09) to 3.2 (0.31) years depending on period of diagnosis and choice of parametric model. The shape of the censoring distribution within intervals did generally not influence results, whereas the choice of parametric model did, even when different models fit the data equally well. In simulation studies both simple midpoint imputation and multiple imputation yielded nearly unbiased analyses (relative biases of -0.6% to 9.4%) and confidence intervals with near-nominal coverage probabilities (93.4% to 95.7%) for censoring intervals shorter than six months. For 12 month censoring intervals, multiple imputation provided better protection against bias, and coverage probabilities closer to nominal values than simple midpoint imputation.

**Conclusion:**

Binning of event and censoring times should be considered a viable strategy for anonymizing register data on survival times, as they may be readily analyzed with methods based on multiple imputation.

## Background

Individualized register data are routinely collected in many countries on a broad variety of diseases, and are becoming an indispensable source of information for health research. However, as informed consent is not obtained from patients, preservation of anonymity is a key concern when allowing researchers access to register data. Often, access is only allowed to binned data in which individuals can no longer be identified. Such data may pose an analytic challenge to researchers since dedicated statistical procedures for this situation are not readily available in standard statistics software, and hence there is a need for general purpose strategies that can be easily implemented in these settings.

The example of binned register data which we will study in this paper arose in the context of colorectal cancer, one of the most frequent malignancies in industrialized countries. Since a substantial proportion of patients either have clinical metastases at the point of diagnosis or develop them in the course of the disease, the prognosis is poor. For Norwegian patients diagnosed in the period 1997-2001 with metastatic rectal cancer (ICD9 C19-21), the survival was limited with a 10.4% 5-year survival for males and 7.8% for females [1]. Corresponding figures for colon cancer (ICD9 C18) were 7.4% for males and 8.6% for females, ibid. In a pivotal study by Hurwitz *et al *[2], patients with metastatic colorectal cancer were randomized to either conventional chemotherapy alone or conventional chemotherapy with addition of bevacizumab. Hurwitz *et al *found a significant treatment effect for adding bevacizumab (hazard ratio for mortality: 0.66, *p *< 0.001).

Consequently, the Norwegian Knowledge Centre for Health Care in spring 2007 was commissioned to undertake a health technology assessment of bevacizumab as guidance for the decision on introducing the drug into standard Norwegian treatment practice. As a key part of the assessment, estimates of recent and current mean survival times in this group of patients were requested, since it was believed that the prognosis had changed over the last two decades and was potentially quite different from those observed in the Hurwitz *et al *study. In 1991, Norwegian physicians' attitude to metastatic colorectal cancer was pessimistic, as only few patients had surgery to remove liver metastases and/or received chemotherapy--and if the latter then 5-fluorourcil only. In recent years, oncologists search more actively for metastases, which are then likely detected earlier; liver metastases are removed if technically possible and the patient is otherwise in good condition; chemotherapy is offered more frequently, and then usually oxaliplatin or irinotecan based.

To preserve anonymity of individual patients, CRN provided counts of deaths and censoring events without any patient specific information grouped into 30 day intervals (termed months in the following). Further, since the clinical trial reported by Hurwitz *et al *excluded patients with a prognosis of less than three months of survival (90 days) [2], it was argued that exclusion of all deaths and loss-to-follow-up events occurring within three months of diagnosis would improve comparability with the clinical trial data. Note, however, that in the clinical trial, deaths did occur also within the first three months, and so the truncation in the Norwegian registry not only removed patients ineligible for treatment with bevacizumab, but inadvertently also those who merely happened to have short survival times and who would otherwise have been eligible.

Although the anonymized data from CRN are interval censored, they are neither of the standard type I or type II interval censored data. Type I is known as current status data, where survival times are only known to be smaller or larger than a given point in time. In type II data, event times are observed to belong to intervals where either both limits are finite and observed, or alternatively one is finite and observed and the other is infinite--see [3] for a detailed discussion. Thus, the monthly counts of deaths are type II interval censored, but the monthly counts of loss-to-follow-up are not, as they do not assign a definitive (left) limit to the interval containing the event. Put differently, the CRN data includes interval censoring of censoring times (loss-to-follow-up). From another perspective, the CRN data may be viewed as an example of life table data with left truncation, but life table data are for non-standard analyses best regarded as interval censored [4]. While it is known that Maximum Likelihood Estimation (MLE) is generally superior to other analytic strategies for interval censored data [5-7], it is not straightforward to conduct a full MLE analysis for this type of data, unless one is willing to adopt restrictive assumptions on the censoring distribution (see below). Using multiple imputation for analyzing interval censored data has been suggested by several, for example [8,9], but not for data with interval censored censorings.

The objective of the present paper is to investigate if a multiple imputation strategy can be utilized to validly fit parametric models to the CRN data. Secondly, it is to develop diagnostic procedures that can be used to assess the fit of any parametric distribution. Finally, to investigate whether mean survival times comparable to those obtained from the clinical trial data by Tappenden *et al *[10] can be reliably estimated, i.e. are reasonably robust to choice of parametric distribution.

The paper is organized as follows: First in the Methods section the CRN data are described and the model is introduced. We then describe a simulation study to assess the validity and statistical efficiency of the developed strategy, develop diagnostic procedures, and introduce a simulation strategy for estimating uncertainty of estimated mean survival times. Parameterization of distributions and details of the implemented strategies are presented at the end of the Methods section. The Results section first reports the results of the simulation study before proceeding to the results of analyzing the CRN data. Finally, results and their implications are discussed.

## Methods

### Material

The Cancer Registry of Norway holds follow-up data on all patients diagnosed with malignancies in Norway in the period 1991-2005. For the present study, data was requested on all patients diagnosed with colorectal cancer (ICD9 codes C18, C19, and C20) for the first time, and where patients at the time of diagnosis had distant metastases (stage 4) and were less than 70 years at diagnosis. Patients who either died or were lost to follow-up earlier than 90 days after diagnosis were excluded, as it was assumed that the remaining patients would be more comparable to those included in the Hurwitz trial [2]. For the included patients, CRN only provided 30 day counts of deaths and loss-to-follow-up, respectively, due to confidentiality concerns. CRN provided data separately for patients diagnosed within each of three time periods (1991-6, 1997-2001, and 2002-5) to allow period specific estimation. All patients were followed until death or Jan 1, 2006, whichever came first. The data provided by CRN are summarized in Table 1, where they have been binned in years for legibility. The full data set is available upon request from the lead author.

**Table 1:** Deaths and censorings among patients with colorectal cancer, Norway, 1991-2005

	1991-1996		1997-2001		2002-2005	
FU-Year	*E*	*C*	*E*	*C*	*E*	*C*
.25	583	0	471	1	360	1
1	393	0	355	3	287	132
2	125	0	143	0	89	97
3	53	0	76	0	33	67
4	33	0	31	0	6	29
5	26	0	19	36		
6	9	0	6	20		
7	9	0	4	25		
8	5	0	2	25		
9	1	0	0	12		
10	3	13				
11	1	13				
12	0	7				
13	0	10				
14	0	7				
15	1	7				
16						

Total	1242	57	1107	122	775	326

Event counts in dataset provided by Cancer Registry of Norway, stratified by period of onset. FU-Year is lower limit of follow-up interval, with the interval extending to the subsequent limit. *E *is count of events, *C *count of censorings. Original data were provided as monthly counts, but is here collapsed into annual counts to improve legibility.

### Ordinary model for survival times

Let *Y *be time to death after diagnosis with survivor distribution function *S_Y _*(**·**; *θ*), density function *f_Y _*(**·**; *θ*), and hazard *h_Y _*(**·**; *θ*), where *θ *is a parameter vector. Let *Z *be a censoring event so that the observable time variable, *X*, is the minimum of *Y *and *Z*, accompanied by an indicator variable denoting which type of event was observed, i.e.:

X= min(Y,Z)andδ=I(X=Y)

If censoring is assumed independent, the marginal likelihood based on all *n *individuals for inference on *θ*, where the censoring contributions have been factored out, looks as follows

(1) $$l\left(\theta ;\left(x,\;\delta \right)\right)=\overset{n}{\underset{i=1}{\prod }}h_{Y}{\left(x_{i};\theta \right)}^{\delta_{i}}S_{Y}\left(x_{i};\theta \right)$$

with **x **= (*x*_1_, *x*_2_, ..., *x_n_*) and *δ *= (*δ*_1_, *δ*_2_, ..., *δ_n_*).

### Model for truncated and interval censored data

In the data provided by CRN, we are not observing from the entire distribution of survival times--events are only observed if they exceed a constant *M *which here is 90 days--and so the likelihood given above in Equation 1 must be modified accordingly. Handling such truncation, or delayed entry as it is, corresponds to considering the conditional distribution of *X *given that *X *≥ *M*. Hence the likelihood becomes

$$l_{X|X\geq M}\left(\theta ;\left(x,\;\delta \right)\right)={\left(S_{Y}\left(M;\theta \right)\right)}^{-1}\overset{n}{\underset{i=1}{\prod }}h_{Y}{\left(x_{i};\theta \right)}_{i}^{\delta }S_{Y}\left(x_{i};\theta \right)$$

Further in the CRN data, all event times, *X*, are subject to interval censoring induced by a sequence of time points, *t*_1_, *t*_2_, ..., *t_m_*_+1_. This means that for each subject only the interval, [*t_j_*; *t_j_*_+1_), and the type of event, *δ*, is observed. Let *n*_1*j *_and *n*_0*j *_be the counts of *X_i_*'s falling within a given period for the two event types, i.e. *n*_1*j *_is the number of deaths and *n*_0*j *_the number of censorings between *t_j _*and *t_j_*_+1_, respectively. If we let *g_j _*be the conditional density of censoring events on the interval [*t_j _*; *t_j_*_+1_) given it occurs in the interval, the likelihood takes the following form when not taking truncation into account

(2) $$l(\theta ;(t,\;n))=\prod_{j=1}^{m}{(S_{Y}(t_{j};\theta )-S_{Y}(t_{j+1};\theta ))}^{n_{1j}}{\left(\int_{t_{j}}^{t_{j+1}}S_{Y}(t;\theta )g_{j}(t)dt\right)}^{n_{0j}}$$

where **t **is the *j ***× **2 matrix with interval end points, *t_j _*and *t_j_*_+1_, as rows, and **n **is the corresponding *j ***× **2 matrix with rows of event counts, *n*_0*j *_and *n*_1*j*._

If events are subject to both truncation and interval censoring the likelihood takes the form

(3) $$l_{X|X\geq M}(\theta ;(t,\;n))=\prod_{j=1}^{m}{\left(\frac{S_{Y}(t_{j};\theta )-S_{Y}(t_{j+1};\theta )}{S_{Y}(M;\theta )}\right)}^{n_{1j}}{\left({\left(S_{Y}(M;\theta )\right)}^{-1}\int_{t_{j}}^{t_{j+1}}S_{Y}(s;\;\theta )g_{j}(s)ds\right)}^{n_{0j}}$$

Equations 2 and 3 implies that for censored events the likelihood involves a term for the censoring distribution, even though censoring is considered to be independent or non-informative. If *g_j _*is non-constant over [t*_j _*; t*_j_*_+1_), then even under an assumption of independent censoring, its shape will be informative when evaluating these contributions as it cannot be factored out. It is thus natural to consider *g_j _*being constant over [*t_j _*; *t_j_*_+1_) as a starting point for analysis, since only this model (the simplest possible) will for most parametric distribution allow direct analytical evaluation of the integrals. For example, for the Weibull survivor function direct analytical evaluation is possible only with uniform *g_j _*'s and then by use of the incomplete Γ-functions. While the time intervals [*t_j _*; *t_j_*_+1_) are all rather short--making the assumption realistic--there are drawbacks to this direct approach. Besides being complex to implement numerically, it more importantly suffers from not allowing the distributional shapes of *g_j _*to be varied easily. Sensitivity analyses can thus not be readily undertaken using this approach. Instead, we suggest to consider this a missing data problem. From this point of view, a simple multiple imputation strategy lends itself naturally to be applied here: Generate for each censored individual *i *a censoring time (*u_il_*) that is independent and distributed according to *g_j _*on the interval wherein censoring is known to have happened--this creates a completed dataset which we label by *l*. This is repeated *k *times to result in *k *completed datasets. The *l*'th dataset can be analyzed by maximizing the following marginal likelihood, where the censoring distribution has been factored out due to the assumption of non-informativeness:

(4) $$l_{X|X\geq M}\left(\theta ;\left(t,\;u,\;n\right)\right)=\overset{m}{\underset{j=1}{\prod }}{\left(\frac{S_{Y}\left(t_{j};\theta \right)-S_{Y}\left(t_{j+1};\theta \right)}{S_{Y}\left(M;\theta \right)}\right)}^{n_{1j}}\underset{\left\{i:\delta_{i}=0\right\}}{\prod }\frac{S_{Y}\left(u_{il};\theta \right)}{S_{Y}\left(M;\theta \right)}$$

**Figure 1:**
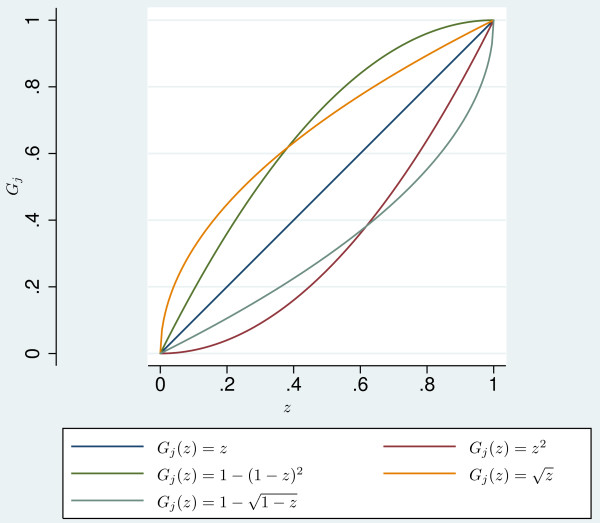
**Censoring distribution functions for sensitivity analysis**. Distribution functions used in the sensitivity analysis on the impact of assuming different shapes of the censoring distribution. *G_j _*is the conditional distribution of *Z *for each of the intervals [*t_j _*; *t_j_*_+1_), *j *= 1, ..., *m*, given that *Z *belongs to the *j*'th interval.

Rubin's formula can be applied to obtain a combined estimate with accompanying uncertainty measures [11], and sensitivity analyses can straightforwardly be conducted by generating *u_il_*'s from various distributions. As distributions we considered those shown in Figure 1, with the diagonal line representing the uniform distribution. Note that the distributions presented in Figure 1 are the conditional distributions on single sub-intervals, i.e. they are not representing the global censoring distribution.

### Simplistic analyses

To allow comparison with simpler analytical strategies, we also present analyses based on single, midpoint imputation (replacing intervals with their midpoint) or ignoring truncation. If all censoring events are replaced by the midpoint of the interval they occur in, but event times are maintained as interval censored, we base the analysis on the following likelihood

(5) $$l_{X|X\geq M}\left(\theta ;\left(t,\;n\right)\right)=\overset{m}{\underset{j=1}{\prod }}{\left(\frac{S_{Y}\left(t_{j};\theta \right)-S_{Y}\left(t_{j+1};\theta \right)}{S_{Y}\left(M;\theta \right)}\right)}^{n_{1j}}{\left(\frac{S_{Y}\left(\frac{t_{j}+t_{j+1}}{2};\theta \right)}{S_{Y}\left(M;\theta \right)}\right)}^{n_{0j}}$$

If also the event times are replaced by the midpoint of the relevant interval, the likelihood becomes

(6) $$l_{X|X\geq M}\left(\theta ;\left(t,\;n\right)\right)=\overset{m}{\underset{j=1}{\prod }}{\left(\frac{f_{Y}\left(\frac{t_{j}+t_{j+1}}{2};\theta \right)}{S_{Y}\left(M;\theta \right)}\right)}^{n_{1j}}{\left(\frac{S_{Y}\left(\frac{t_{j}+t_{j+1}}{2};\theta \right)}{S_{Y}\left(M;\theta \right)}\right)}^{n_{0j}}$$

To ignore truncation, the term *S_Y _*(*M*)^-1 ^is removed from the relevant likelihood.

### Simulation study

To investigate the statistical properties of the multiple imputation strategy developed above, we set up a simulation study in which we compared the multiple imputation strategy with a simple, single imputation of interval midpoints for interval censored censoring events (Equation 5 above). We generated event times as Weibull distributed (*S_Y _*(*t*; *λ, γ*) = exp(-*λy^γ^*)), where we as true values of the parameters used those estimated for the last period of the Norwegian registry data (log λ = -0.5902 and γ = 0.9425, cf. results below). Censoring was taken to be non-informative and occurring with constant rate corresponding to annual proportions of 3%, 6%, and 9%, respectively. All event times were censored at the end of follow-up, which occurred at ten years. We varied the width of censoring intervals (1, 2, 6, and 12 months, where each month is 30 days) and the sample size (500, 1,000, and 10,000). In each of the 36 settings we analyzed 2,500 generated datasets. For each setting we report the median relative bias (the difference between the median of estimates and the true value relative to the true value), the empirical coverage probability of 95% confidence intervals, and the inflation factor of standard errors. The latter is defined as the ratio between the median standard error obtained with interval censored data and the median of standard errors obtained from analysis of identical data that have not been subjected to interval censoring, but only to ordinary right censoring. Note, that the performance of the method is not evaluated under misspecification (in practical applications misspecification should be dealt with using the tools developed below) as the focus is here only on studying the ability to handle interval censoring.

### Goodness-of-fit test

To conduct valid parametric analyses it is essential to have access to diagnostic procedures that allow assessing the fit of the chosen parametric family. As the data under study are already binned, we propose a variant of the χ^2 ^goodness-of-fit test that accommodates censoring.

The expected counts can be derived from considering the following density, evaluated at the fitted parameter values:

(7) $$f_{Y|Y\leq Z,Y\geq M}\left(t;\hat{\theta }\right)=\frac{\int_{t}^{\infty }f_{\left(Y,Z\right)}\left(t,u;\hat{\theta }\right)du}{\int_{M}^{\infty }\int_{z}^{\infty }f_{\left(Y,Z\right)}\left(t,u;\hat{\theta }\right)du\;dz}$$

where $f_{\left(Y,Z\right)}\left(.,\;\cdot ;\hat{\theta }\right)$ is the joint density of times of death and censoring, *Y *and *Z*, evaluated at the maximum likelihood estimate $\hat{\theta }$. Since *Y *and *Z *are independent, this can be written as

$$f_{Y|Y\leq Z,Y\geq M}\left(t\right)=\frac{f_{Y}\left(t;\hat{\theta }\right)S_{Z}\left(t\right)}{\int_{M}^{\infty }f_{Y}\left(z;\hat{\theta }\right)S_{Z}\left(z\right)dz}$$

While *f_Y _*was estimated above, we now additionally need an estimate of the censoring distribution, specifically *S_Z_*. Had *Y *and Z not been interval censored, it would have been straightforward to get an estimate of *S_Z _*via Kaplan-Meier with deaths considered as censoring events and censorings as failure events. We thus propose to impute *Y *and *Z *and then estimate *S_Z _*by use of the Kaplan-Meier estimator, before computing expected values for each interval by averaging over imputations. Specifically, we suggest the following algorithm for generating imputed datasets:

1. For each observed event *Y_i _*in interval [*t_j _*; *t_j_*_+1_), the event time is imputed from the fitted survival function $f_{Y}\left(\cdot ;\hat{\theta }\right)$ conditional on *Y *∈ [*t_j _*; *t_j_*_+1_).

2. For each censoring event *Z_i _*in interval [*t_j_*; *t_j_*_+1_), the time of censoring is imputed from a uniform distribution over this interval.

3. From the imputed dataset, the Kaplan-Meier estimate of *S_Z _*is found by considering loss-to-follow-up as event times and deaths as censoring events.

4. From the fitted distributions for *Y *and *Z*, the density in Equation 7 is numerically integrated over each relevant time interval. The expected count for each interval is found by multiplication with the total number of observed events.

Steps 1 to 4 are repeated to create 10 imputed datasets with expected counts. The overall expected count is then found by averaging over the imputed datasets.

Finally, the χ^2 ^goodness-of-fit test statistic was computed as follows: If the expected number of events in an interval was smaller than five, the interval was joined with its lower neighbor. This procedure was repeated until all expected values were greater than five. The goodness-of-fit test statistic was evaluated as

$$\chi^{2}=\overset{\tilde{m}}{\underset{\tilde{j}=1}{\sum }}\frac{{\left(O_{\tilde{j}}-\text{E}\left(N_{\tilde{j}}\right)\right)}^{2}}{\text{E}\left(N_{\tilde{j}}\right)}$$

where $\tilde{j}$ and $\tilde{m}$ are the index and count of joined intervals, respectively, and $O_{\tilde{j}}$ is the observed count of events in the $\tilde{j}$'th interval. The test statistic was evaluated in the χ^2 ^distribution with $\tilde{m}-q-1$ degrees of freedom, where *q *is the number of parameters fitted in the parametric model.

### Estimation of mean survival time

For all the distributions analyzed in this paper (see Table 2), the mean survival could be computed directly from the estimated parameters, except for the Gompertz distribution. The Gompertz estimates did however correspond to infinite mean survival, and so no alternative, numerical strategy was needed in this case. To reflect the uncertainties of the estimated parameters in computed means, we used a simulation strategy since this avoids the assumption of normality of the estimated mean and the assumption of differentiability, both of which are required by the delta method. For each parameter set, we thus sampled 10,000 times from a multivariate normal distribution defined by the estimate and their estimated covariance matrix, and for each sampled value computed the corresponding mean. As overall estimate of the mean survival time we used the median of the means accompanied by an empirical 90% confidence interval, i.e. the 5% and 95% percentiles in the sampled distribution of means.

**Table 2:** Parameterization of distributions

Model	*S*(*y*)	Parameter restrictions	Mean
Weibull:	exp (-*λy^γ^*)	*λ *> 0, *γ *> 0	$\frac{\Gamma \left(1+\frac{1}{\gamma }\right)}{\lambda^{1∕\gamma }}$
Gamma:	$I{\left(\alpha ,\;\frac{y}{\beta }\right)}^{†}$	*α *> 0, *β *> 0	*αβ*
Gompertz:	$\text{exp}\left(-\frac{\lambda }{\gamma }\left(e^{y\gamma }-1\right)\right)$	*γ *> 0	No closed formula
Log-Logistic:	$\frac{1}{1+{\left(y∕\alpha \right)}^{\beta }}$	*α *> 0, *β *> 0	$\frac{\alpha \pi }{\beta sin\left(\pi ∕\beta \right)}$
Log-Normal:	$\Phi {\left(-\frac{log\left(y\right)-\mu }{\sigma }\right)}^{\text{‡}}$	*σ *> 0	$\text{exp}\left(\mu +\frac{1}{2}\sigma^{2}\right)$

^† ^*I*(*a, x*) is the incomplete gamma function given by

$$I\left(a,\;x\right)=\frac{1}{\Gamma \left(a\right)}\int_{0}^{x}e^{-t}t^{a-1}dt$$

^‡ ^Φ(x) is the standard normal cdf given by

$$\Phi \left(x\right)=\frac{1}{\sqrt{2\pi }}\int_{-\infty }^{x}e^{-\frac{1}{2}t^{2}}dt$$

For each distribution the survivor function *S*(*y*) is given accompanied by parameter restrictions, if applicable, and the formula for the mean survival as a function of parameters.

### Implementation

The parametric distributions considered and their parameterization are shown in Table 2. All analyses were conducted using Stata 9 [12]. More specifically, the likelihoods in Equations 1 and 4, respectively, were both coded and evaluated using the general maximum likelihood command, -ml-, available in Stata 9, cf. [13]. The multiple imputation used the -micombine- command of the ICE add-on package to obtain joint estimates across imputations [14]. Throughout we have chosen the number of imputations to be 10 [11, p. 114]. Examples of code used for the computations are available upon request to the authors.

## Results

### Simulation results

Table 3 presents relative bias, coverage probabilities and inflation of standard errors when either using single midpoint imputation or multiple imputation for interval censored censoring events. When interval censoring is induced by intervals with lengths up to six months, both analytic strategies perform well with negligible bias, coverage probabilities of confidence intervals close to the nominal value, and standard errors that are only marginally increased relative to the ordinary situation, i.e. when only ordinary right censoring is present. As a general tendency, however, the multiple imputation has lower relative bias and better coverage, in particular when censoring becomes more dominant in terms of higher censoring rates and wider censoring intervals. Only in extreme cases of 6% and 9% annual censoring proportions, censoring intervals of one year length, and larger sample sizes of 10,000, does coverage probabilities of the multiple imputation strategy decrease unacceptably to levels around 75% to 80%, albeit not as low as when single midpoint imputation is used. This poor performance in extreme cases is to be expected, as the conditional censoring distribution is taken to be uniform in the multiple imputation analysis, while censoring times are actually generated from an exponential model--it is this effect that becomes apparent when datasets become large and intervals very wide, but not before. In all situations, both analytic strategies yield virtually identical statistical efficiency, which depend mostly on the width of censoring intervals, and less on the rate of censorings. The loss in statistical efficiency is twice as high for the shape parameter γ as for the scale parameter log(λ).

**Table 3:** Simulation results comparing analyses with multiple imputation or single midpoint imputation in terms of bias, coverage probability and relative efficiency

				1 month			2 months			6 months			12 months		
Parameter	Censoring	Estimation	*n*	*RB*	*Cov*	*SEIF*	*RB*	*Cov*	*SEIF*	*RB*	*Cov*	*SEIF*	*RB*	*Cov*	*SEIF*
log(λ)	3%	MC	500	3.0	95.5	0.8	-3.2	94.9	1.6	7.8	94.9	6.4	17.7	94.7	16.8
			1,000	0.0	94.7	0.8	3.5	94.3	1.7	2.6	95.6	6.4	14.5	94.9	16.7
			10,000	0.4	95.4	0.8	-0.3	94.9	1.7	2.9	95.5	6.4	14.2	91.0	16.8
		
		MI	500	3.0	95.5	0.8	-3.4	94.9	1.6	5.7	94.9	6.4	11.1	95.0	16.8
			1,000	-0.1	94.8	0.8	3.3	94.3	1.7	1.0	95.4	6.4	8.2	94.8	16.7
			10,000	0.3	95.4	0.8	-0.3	94.9	1.7	1.3	95.6	6.4	8.0	94.0	16.8
	
	6%	MC	500	2.3	95.7	0.7	0.6	94.7	1.6	9.4	95.4	6.3	29.6	94.0	16.6
			1,000	0.4	94.8	0.7	1.2	95.5	1.6	9.1	95.7	6.3	29.6	94.0	16.7
			10,000	1.1	94.7	0.8	0.7	94.6	1.7	9.0	94.0	6.3	30.7	77.6	16.8
		
		MI	500	2.2	95.7	0.7	0.4	94.7	1.6	6.9	95.2	6.3	19.7	94.6	16.8
			1,000	0.3	94.7	0.7	1.1	95.5	1.6	7.2	95.7	6.3	21.3	94.6	16.9
			10,000	1.0	94.7	0.8	0.5	94.6	1.7	6.8	95.0	6.4	22.5	85.7	17.0
	
	9%	MC	500	0.4	95.4	0.8	-0.9	95.3	1.6	11.1	95.4	6.2	49.7	92.8	16.9
			1,000	0.6	95.4	0.7	0.9	95.4	1.5	12.2	95.0	6.2	46.7	91.8	16.8
			10,000	0.7	95.1	0.8	1.5	94.9	1.6	12.2	91.9	6.2	45.7	57.1	16.8
		
		MI	500	0.3	95.4	0.8	-0.9	95.4	1.6	8.3	95.4	6.3	39.3	93.8	17.2
			1,000	0.6	95.4	0.7	0.5	95.4	1.5	9.5	95.1	6.3	35.9	93.3	17.1
			10,000	0.5	95.1	0.8	1.2	94.8	1.6	9.4	93.4	6.3	34.9	73.7	17.1

γ	3%	MC	500	0.6	95.0	2.0	1.0	94.8	4.1	3.9	94.5	13.5	7.6	94.8	30.2
			1,000	0.8	95.4	2.1	2.1	95.1	4.4	1.6	95.0	13.6	6.9	95.0	30.3
			10,000	-0.2	95.3	2.0	0.5	95.1	4.2	1.6	94.9	13.6	6.0	90.7	30.3
		
		MI	500	0.4	94.8	2.0	0.9	94.8	4.1	2.0	94.5	13.4	2.6	94.8	29.8
			1,000	0.6	95.4	2.1	2.0	95.0	4.4	-0.0	94.8	13.5	2.1	95.0	29.9
			10,000	-0.4	95.2	2.0	0.4	95.2	4.2	-0.1	95.1	13.4	1.2	94.4	29.8
	
	6%	MC	500	2.4	95.7	2.0	1.7	94.6	4.1	6.4	94.8	14.1	12.8	94.1	31.6
			1,000	-0.4	95.5	2.0	1.8	95.4	4.4	4.5	94.6	14.1	13.3	93.9	31.8
			10,000	0.1	94.3	2.2	0.7	94.6	4.5	3.7	92.9	14.3	12.8	79.8	31.9
		
		MI	500	2.2	95.6	2.0	1.5	94.6	4.1	4.1	94.9	14.0	6.8	94.4	31.3
			1,000	-0.6	95.5	1.9	1.7	95.4	4.5	2.6	94.4	14.1	7.3	94.8	31.6
			10,000	-0.0	94.2	2.2	0.6	94.6	4.5	1.8	94.2	14.2	6.8	91.3	31.7
	
	9%	MC	500	3.9	95.6	2.6	3.0	95.7	4.7	6.1	95.4	15.2	20.3	92.4	33.8
			1,000	1.9	95.5	2.2	1.8	94.4	4.7	7.2	95.4	15.0	21.0	92.3	33.6
			10,000	-0.1	95.5	2.3	1.1	95.0	4.7	5.4	92.4	15.0	20.6	57.1	33.6
		
		MI	500	3.7	95.6	2.6	2.8	95.7	4.7	3.5	95.6	15.2	12.9	93.5	33.7
			1,000	1.7	95.6	2.2	1.6	94.4	4.7	4.8	95.4	15.0	13.3	94.6	33.5
			10,000	-0.3	95.4	2.3	0.9	95.2	4.7	3.2	94.2	15.0	12.9	80.4	33.5

Simulation results for estimation of log(λ) and γ from datasets with Weibull distributed event times, constant censoring rates, ten years of follow-up, and varying widths of interval censorings. Results for each setting represent analyses of 2,500 generated datasets. Column headers in months indicate width of intervals inducing interval censoring; *censoring *refers to annual proportion of censoring; *Estimation *refers to estimation procedure: **MC **to single imputation of midpoint for censoring events (Equation 5) **MI **to multiple imputation, (Equation 4). *RB *is median relative bias in percent, *Cov *is coverage probability of nominal 95% confidence intervals, while *SEIF *is percent increase in median standard error relative to analysis with ordinary censoring, but no interval censoring.

### Application results

**Figure 2:**
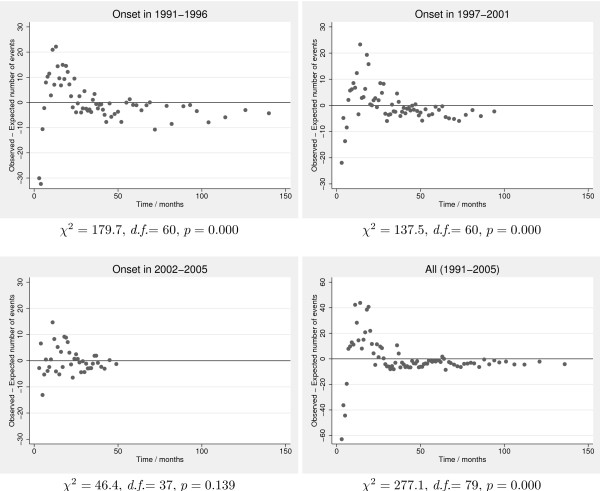
**Goodness-of-fit plots**. Difference between expected and observed counts of events over the time scale. *χ*^2 ^is the goodness-of-fit test statistic value with *d.f*. degrees of freedom and associated *p*-value. Note that the plot for all periods pooled has a differently scaled Y-axis than the period specific plots.

All the five fitted distributions (Gamma, Gompertz, Log-Logistic, Log-Normal, and Weibull) fitted the data well in the last period (2002-2005), cf. Table 4. None of the distributions achieved adequate fit for the other periods, including when all data was pooled, although the Gompertz according to *p*-values and diagnostic plots came close for the first period, 1991-1996 (plots not shown, available upon request). Figure 2 displays diagnostic plots for the Weibull distribution in each of the three periods separately and when pooled, which supports that the fit was adequate only in the last period, 2002-2005. In the other periods, the number of events is generally overestimated in the beginning, then underestimated from six months to approximately three years, and finally overestimated at the right tail. As the last period has a shorter follow-up period, the fit of any parametric model is only determined by the events in the earlier part of the follow-up period. Plots for the other distributions are available upon request.

**Table 4:** Goodness-of-fit tests and estimated mean survival times for five parametric distributions, patients with metastatic colorectal cancer, Norway 1991-2005

Period	Model	$\chi_{\text{ob}1\text{s}}^{2}$	*d.f.*	*p*	Mean	90% CI
1991-1996	Gamma	225.1	62	0.0000	-^†^	-
	Gompertz	75.9	55	0.0324	**∞**	-
	Log-Logistic	81.3	56	0.0151	2.29	(2.06; 2.58)
	Log-Normal	116.9	58	0.0000	1.61	(1.49; 1.75)
	Weibull	179.7	60	0.0000	1.19	(1.06; 1.34)
	
1997-2001	Gamma	154.2	60	0.0000	1.25	(0.91; 1.50)
	Gompertz	79.2	53	0.0114	**∞**	-
	Log-Logistic	80.8	55	0.0135	3.20	(2.80; 3.79)
	Log-Normal	98.2	56	0.0004	2.06	(1.92; 2.22)
	Weibull	137.5	60	0.0000	1.71	(1.56; 1.87)
	
2002-2005	Gamma	46.9	36	0.1053	1.89	(1.77; 2.02)
	Gompertz	44.5	35	0.1312	**∞**	-
	Log-Logistic	43.1	34	0.1358	3.14	(2.76; 3.65)
	Log-Normal	45.3	35	0.1142	2.39	(2.24; 2.54)
	Weibull	46.4	37	0.1392	1.92	(1.81; 2.05)
	
All	Gamma	319.7	81	0.0000	1.23	(1.06; 1.38)
	Gompertz	135.8	70	0.0000	**∞**	-
	Log-Logistic	127.5	74	0.0001	2.87	(2.67; 3.11)
	Log-Normal	170.0	77	0.0000	2.01	(1.93; 2.09)
	Weibull	277.1	79	0.0000	1.64	(1.55; 1.73)

^† ^Could not be meaningfully estimated due to unreliable parameter estimates (the estimated standard error of the *α *parameter approached infinity).

Goodness-of-fit *χ*^2 ^test statistics, degrees of freedom (*d.f*.), and *p*-value is given for each period and all data pooled. The mean survival times are based on 10,000 draws from a bivariate normal distribution given by the estimated parameters and their covariance. For each draw a mean is computed with the appropriate formula given in Table 2, and the median together with 5% and 95% percentiles of this posterior distribution are used as estimate and confidence interval, respectively.

The estimated means varied substantially between the distributions, both when the fit was poor (long follow-up time, 1991-1996), and when the fit was good (shorter follow-up time, 2002-2005). The means ranged from 1.89 years (Gamma) to 3.14 years for the Log-Logistic and even infinity for the Gompertz in the last period, 2002-2005 (Table 4). The likelihood ratio test for homogeneity of Weibull distributions across periods showed statistical significance with a χ^2 ^value of 122.7 on 4 degrees of freedom yielding *p *≪ 0.0001. Note, that while mean survival times varied between periods, the direction of change was not consistent from distribution to distribution: For the Weibull, Log-Normal, and the Gamma distribution mean survival time increased with period, the Gompertz found it to be infinite in all periods, whereas the Log-Logistic suggested an increase between the first two periods and then a small decline for the last. Both the Gamma and Log-Logistic distributions have asymmetric confidence intervals for the mean, something that would not easily have been detected if we had used the delta method for estimating means from the parameter estimates. Based on the trial by Hurwitz *et al *[2], the mean survival was estimated to 1.98 years in the intervention group and 1.57 years in the control group by Tappenden *et al *[10].

For the Weibull distribution we estimated parameters using different approaches for handling the interval censoring and truncation, cf. Table 5. As expected from the simulation study above, the use of single midpoint imputation for censoring events in this situation yields virtually identical estimates to those based on multiple imputation--a consequence of the short censoring intervals. Results based on imputing midpoints of intervals for both events and censoring differ slightly more, but are for all practical purposes still identical. Ignoring the truncation does however create noticeably bias in the estimated mean survival time.

**Table 5:** Weibull Parameter estimates and estimated mean survival times, patients with metastatic colorectal cancer, Norway 1991-2005

Period	Estimation	$\hat{\text{log}\left(\lambda \right)}$ (s.e.)	$\hat{\gamma }$ (s.e.)	Mean	**s.e**.	Median	5%	95%
1991-1996	MI	0.2662 (0.0669)	0.4945 (0.0287)	1.196	0.086	1.194	1.057	1.340
	MC	0.2662 (0.0669)	0.4944 (0.0287)	1.196	0.086	1.194	1.057	1.340
	MA	0.1982 (0.0799)	0.3741 (0.0256)	1.203	0.086	1.201	1.064	1.346
	NT	-0.6520 (0.0358)	0.9200 (0.0185)	2.116	0.065	2.115	2.011	2.223
	
1997-2001	MI	-0.0700 (0.0679)	0.5962 (0.0334)	1.709	0.095	1.706	1.556	1.867
	MC	-0.0700 (0.0679)	0.5962 (0.0334)	1.709	0.095	1.706	1.556	1.867
	MA	-0.1752 (0.0685)	0.5801 (0.0319)	1.713	0.094	1.710	1.561	1.871
	NT	-0.8462 (0.0399)	1.0051 (0.0230)	2.317	0.071	2.314	2.204	2.435
	
2002-2005	MI	-0.5902 (0.0664)	0.9425 (0.0516)	1.926	0.074	1.925	1.806	2.051
	MC	-0.5904 (0.0664)	0.9428 (0.0516)	1.925	0.074	1.925	1.806	2.050
	MA	-0.6118 (0.0659)	0.9506 (0.0509)	1.926	0.074	1.925	1.807	2.050
	NT	-1.0586 (0.0467)	1.3282 (0.0386)	2.043	0.057	2.042	1.952	2.138
	
All	MI	-0.0650 (0.0385)	0.6099 (0.0197)	1.641	0.052	1.641	1.555	1.728
	MC	-0.0650 (0.0385)	0.6099 (0.0197)	1.641	0.052	1.641	1.555	1.728
	MA	-0.0722 (0.0424)	0.5048 (0.0178)	1.645	0.052	1.645	1.559	1.731
	NT	-0.8148 (0.0229)	1.0132 (0.0133)	2.224	0.040	2.223	2.159	2.291

Weibull parameter estimates for the three observation periods for survival after colon-rectal cancer based on data from the Cancer Registry of Norway. Estimated mean survival time for each of the three periods. Mean, s.e., median, 5%, and 95% percentiles all refer to the distribution of computed means obtained when sampling from the distribution of estimates. All subjects with event times smaller than three months are excluded by design. **MI **is multiple imputation (Equation 4), **MC **means replacing censored observations with the midpoint of their interval (Equation 5), **MA **means replacing all events and censored observations with the midpoint of their interval (Equation 6), **NT **means multiple imputation, but ignoring the truncation (Equation 4 with the term *S_Y _*(*M*)^-1 ^omitted).

Table 6 gives the results of applying the four different assumptions regarding the shape of the censoring distribution within each interval of one month length when using a Weibull distribution for event times. We only give results for the last time period and for all three time periods joined together, but results for the other time periods (not shown) were similar in the sense that changes in estimates were negligible with respect to choice of censoring distribution. This is a result of the censoring intervals being short in this case (one month), but in applications with longer intervals we would suggest mimicking this sensitivity analysis, as the assumed shape of censoring distribution becomes more important with long intervals.

**Table 6:** Results of sensitivity analysis with respect to the assumed shape of the interval specific censoring distribution

	2002-2005		All	
*G_j_*(*z*)	$\hat{\text{log}\left(\lambda \right)}$ (s.e.)	$\hat{\gamma }$ (s.e.)	$\hat{\text{log}\left(\lambda \right)}$ (s.e.)	$\hat{\gamma }$ (s.e.)
z	-0.5902 (0.0664)	0.9425 (0.0516)	-0.0650 (0.0385)	0.6099 (0.0197)
*z*^2^	-0.5884 (0.0666)	0.9383 (0.0516)	-0.0642 (0.0386)	0.6090 (0.0197)
1 - (1 - *z*)^2^	-0.5924 (0.0663)	0.9473 (0.0517)	-0.0658 (0.0385)	0.6109 (0.0197)
$\sqrt{z}$	-0.5926 (0.0663)	0.9475 (0.0517)	-0.0659 (0.0385)	0.6109 (0.0197)
$1-\sqrt{1-z}$	-0.5882 (0.0666)	0.9381 (0.0516)	-0.0642 (0.0386)	0.6090 (0.0197)

Weibull parameter estimates for the period 2002-2005 and all periods joined together, respectively, under four different assumptions regarding the shape of the censoring distribution on each interval, *G_j_*.

## Discussion

The analyses above showed how a multiple imputation strategy combined with a relatively simple maximum likelihood estimation procedure could yield a flexible and valid parametric analysis of interval censored data, and at the same time avoid numerical complexities. Based on the estimated parameters, mean survival times and their uncertainty could be estimated, and finally a goodness-of-fit test was implemented by utilizing the inherent binning of the interval censored data together with a multiple imputation strategy. The simulation studies demonstrated that as long as intervals are not too wide, the multiple imputation is a valid analytic strategy with low loss of statistical efficiency relative to analyses of data without interval censoring.

In the simulation study we focused attention on the statistical properties of the imputation strategy when the parametric model is correctly specified. As shown in our analysis of the Norwegian register data, it is however often not simple in actual applications to identify which model is correctly specified--if such a tractable model exist at all--even with adequate statistical diagnostic procedures. While the problem is not restricted to binned data, it may become attenuated by the binning, as it may make the detection of deviations from the assumed distribution more difficult. If this aspect should have been studied in a simulation study, one might suggest to compare the results of misspecified analyses based on ordinary right censored data and binned data, respectively. We are however confident that only when intervals become wide will the problem of misspecification have the potential to become more pronounced than in the ordinary right censored situation, as for short intervals results are virtually identical between analyses based on right censored and binned data. Our simulation study shows that the imputation method should with wide intervals be used cautiously anyway, as the bias is then large even when the model is correctly specified.

While the CRN data were truncated and interval censored, it might be argued that both features were not prominent for the data: Only the first 90 days of data are discarded, and the binning in 30 day intervals is but a fine grained filtering. Even though three months is short compared to the length of follow-up--in particular for the earlier cohorts--we showed that three months is considerable with respect to estimated means, and this aspect of the data can thus not be ignored. The simulation studies revealed that the impact of interval censoring was generally less important, especially for narrow intervals. Even so, the simulation studies revealed that while single midpoint imputation yielded identical results for narrow intervals, the suggested multiple imputation strategy provided better protection against bias and confidence intervals with coverage probabilities closer to nominal values in situations with wider censoring intervals. Although both deaths and censoring events were interval censored in the CRN data, multiple imputation was only done with respect to censoring events in the estimation process. The rationale was that the distribution of deaths was predetermined by a parametric distribution, and so their likelihood contributions were given and straightforward to calculate. The censoring distribution on the other hand is not itself of interest, and so the focus here is on sensitivity of main parameter estimates to different choices of the distribution of censoring. If the interest had been in conducting semi-parametric estimation (Cox regression) of the event distribution, then multiple imputation for events (deaths) might be a simple alternative to dedicated methods for interval censored data.

Finally, it should be noted that although cost-effectiveness analyses may mandate estimation of mean survival times, this should not be considered a trivial endeavor, see for example [15]. In most studies involving survival times, the right tail of the distribution is unobserved due to right censoring, and yet this tail is highly influential on the mean--even in situations where only a small proportion survives past the end-of-follow-up and different parametric distributions fit equally well, as documented in our analyses above. It is for this reason that the mean restricted to the observation period is commonly used in cost-effectiveness analyses, although it makes comparisons with studies with different lengths of follow-up impossible. The estimated mean from a parametric model will necessarily depend on the specific shape implicitly assumed for the unobserved part of the distribution, and so the sensitivity of the mean to distributional assumptions should be explored whenever possible, as we have done here.

## Conclusion

Provision of binned data to maintain anonymity of patients should be considered a viable procedure, since a multiple imputation strategy can be used to account for the interval censoring created by the binning, as long as intervals are not too wide.

## Competing interests

The authors declare that they have no competing interests.

## Authors' contributions

HS and ISK jointly developed the original idea for the study. HS developed the statistical analyses and conducted them, and drafted the original and revised manuscripts. ISK provided access to data. The interpretation of data analyses was the product of discussions between the authors. ISK commented on drafts of the paper. Both authors have seen and approved the final version.
